# Hierarchical based classification method based on fusion of Gaussian map descriptors for Alzheimer diagnosis using T_1_-weighted magnetic resonance imaging

**DOI:** 10.1038/s41598-023-40635-2

**Published:** 2023-08-23

**Authors:** Shereen E. Morsy, Nourhan Zayed, Inas A. Yassine

**Affiliations:** 1 Systems and Biomedical Engineering, Cairo University, Cairo, Egypt; 2 Computer and Systems Department, Electronics Research Institute, Cairo, Egypt; 3 Mechanical Engineering Department, The British University in Egypt, Cairo, Egypt

**Keywords:** Biomarkers, Biomedical engineering, Neurological disorders

## Abstract

Alzheimer’s disease (AD) is considered one of the most spouting elderly diseases. In 2015, AD is reported the US’s sixth cause of death. Substantially, non-invasive imaging is widely employed to provide biomarkers supporting AD screening, diagnosis, and progression. In this study, Gaussian descriptors-based features are proposed to be efficient new biomarkers using Magnetic Resonance Imaging (MRI) T_1_-weighted images to differentiate between Alzheimer’s disease (AD), Mild Cognitive Impairment (MCI), and Normal controls (NC). Several Gaussian map-based features are extracted such as Gaussian shape operator, Gaussian curvature, and mean curvature. The aforementioned features are then introduced to the Support Vector Machine (SVM). They were, first, calculated separately for the Hippocampus and Amygdala. Followed by the fusion of the features. Moreover, Fusion of the regions before feature extraction was also employed. Alzheimer's disease Neuroimaging Initiative (ADNI) dataset, formed of 45, 55, and 65 cases for AD, MCI, and NC respectively, is appointed in this study. The shape operator feature outperformed the other features, with 74.6%, and 98.9% accuracy in the case of normal vs. abnormal, and AD vs. MCI classification respectively.

## Introduction

One of the chronic progressive neurodegenerative diseases is AD. Alzheimer’s Disease is the sixth cause of mortality in the US in 2015^1^. Across the broad, the people living with AD are nearly 44 million. More and above, in the next two decades, the estimated number of affected people will double^2^. So that by 2050, one out of 85 persons will have AD^2^. It is considered a dementia disease that is linked with some behavioral alterations and memory leakage because of the death of brain cells^3^. Manifestations of early Alzheimer’s attack start between the age of thirty’s and sixtieths. The first manifestations vary from patient to patient. Memory problems are often one of the first cognitive impairment signs. As the disease progresses, people may be diagnosed with Mild Cognitive Impairment (MCI), since they experience greater memory loss as well as other cognitive difficulties. In this stage, patients may conduct their commonplace stirrings but with minimal adequacy. MCI is considered the longest stage, as it may last for 20–30 years. AD progresses in sundry stages: preclinical, mild (at times named early-stage), moderate, and severe (at times named late-stage). The late stage of the disease where patients may stay for 5 years, usually ended with patient death^4^.

Since AD averages are predictable to dramatically increase in the upcoming years. Though, its early diagnosis and treatment act as an essential confront in modern science. In addition, the advances in neuroimaging and finding new biomarkers attempts are changing our understanding of AD. Structured magnetic resonance imaging (MRI) has a great range of soft tissue contrast that can describe the anatomy in detail, a new biomarker-based definition using MRI can measure neuro-degeneration^4,5^. Early research in the MRI images analysis for AD patients is focused on estimating the brain/region atrophy (or brain/region volumes)^6–8^, quantifying the MRI signal alterations due to the changes in tissue characteristics such as white matter hyperintensities from T_2_-weighted images^9^. Damulina et al.^10^ calculated WMH load in different brain regions, comparing normal control subjects to AD patients confirmed elevated WMH load in AD patients, especially in the brain regions periventricular, parietal white matter, and subcortical frontal brain. Others emphasize atrophy, of the hippocampus and nearby medial temporal structures, by analyzing the regional volumetric changes in patients with AD compared to normal control subjects^6–8,11^.

From the authors’ readings, previous work established approaches, to automatically classify the different stages of AD, which can be assorted into triple categories: voxel-based approaches, vertex-based approaches, and approaches using Region of Interest (ROI) based. In the approaches using voxels, the features are representing the probability maps of the tissue type in each voxel. The probability map is usually calculated through the histogram of intensities estimation of each voxel^9,12–15^. In the case of vertex-based approaches, the features are usually extracted from the cerebral cortical surface such as the cerebral cortex thickness at each vertex of the cortex. The inner and outer surface of the cortex is extracted and represented as the same number of vertices, where further measures are extracted such as the cortical thickness (defined as the Euclidean distance between these linked vertices)^16–18^. When doing the exploration based on the volume it is called ROI-based approaches^15,19^, Other ROI way is by exploring the shape of the most affected regions due to the progression of the disease^16,20^. A recent study put together a complex volumetry-based analysis and vertex-based analysis to investigate the pattern of subregional structural changes in gray matter structures and correlated this with the clinical scores for AD, and MCI, compared to the normal controls. Their results showed significant atrophy in bilateral hippocampi and nucleus accumbens for AD patients compared to normal controls^8^.

The Hippocampus and Amygdala are considered the utmost smitten part in terms of shape by Alzheimer’s retrograding)^6,13,20^. Adding up the hippocampus important role associated with long-term memory, which includes all bygone awareness, and experiences^21–23^. Whereas, the Amygdala plays a pivotal function in the emotional processing such as: memory associated with emotions, and emotional stimuli adaptive responses^24^. Thus, finding new biomarkers is changing our understanding of AD, especially zooming out on these regions associated with cognitive impairment of patients, which might be confirming them to be potential target regions of treatment in AD. Otherwise, the existing challenge for modern neuroimaging is to help diagnose early AD and MCI patients. This will reflect the disease stage as well as the predictive progression of mild cognitive.

This paper employs the Gaussian Map based features, extracted from ROI to distinguish between the normal controls, MCI, and AD patients. The Gaussian Map features were extracted for both the Hippocampus and Amygdala separately once and once again after contaminating the two regions to study the overall changes in the shape than studying this for each region separately. For more assessment and understanding, two fusion levels were done; Feature-fusion level which was done by calculating independently each ROI extracted feature and then fusing both for further classification. ROI-fusion level whereas fusion of the regions before feature extraction was also employed. The classification process is following hierarchical criteria where, in the first phase, the objective is to discriminate NC from the abnormal subjects; named AD and MCI. The second step is to differentiate the AD and MCI subjects. The Gaussian-based extracted features were then given to the SVM classifier. Finally, the performance of the proposed extracted features will be evaluated calculating sensitivity, specificity, accuracy, and ROC Analysis^25^.

## Materials and methods

### Dataset description

Our study includes one hundred sixty-five subjects (165), Tables 1 and 2 list the age range of the subjects employed in the study as well as the inclusive diagnostic criteria such as Mini-Mental State Exam score (MMSE) Clinical Dementia Rating (CDR). The dataset was acquired using the ADNI acquisition protocol, high resolution selected volumes were acquired in the transverse plane by a 3-Tesla MRI scanner, and a 3D MPRAGE T_1_-weighted sequence. Table 3 shows all the scanning parameters. A complete description of ADNI is available at http://adni.loni.usc.edu/. The enrolled normals’ and patients’ age ranged from fifty-six years old to eighty (inclusive) years old, who at least completed six grades of education (or had a good work history sufficient to exclude mental retardation) as shown in Table 1. All subjects will have clinical/cognitive assessments and structural MRI scan at specified intervals for two to three years. Approximately 50% of subjects will also have PET scans at the same time intervals and 25% of subjects (who have not been scanned using PET) will have MRI at 3 T.

**Table 1:** Dataset breakdown age and gender.

	Male	Female	Count/age range
Normal	28 Subjects	37 Subjects	65 Subjects
	Age: 64–76 year	Age: 70–80 year	64–80 year
MCI	29 Subjects	26 Subjects	55 Subjects
	Age:60–80	Age:56–79	56–80 year
AD	24 Subjects	21 Subjects	45 Subjects
	Age: 57–79 year	Age: 57–78 year	57–79 year

**Table 2:** Dataset inclusion guidelines.

Dataset	ADNI	MMSE scores (inclusive)	CDR (inclusive)	Other conditions (inclusive)
AD	45 subjects	20–26	0.5 or 1.0	Meets NINCDS/ADRDA criteria for probable AD
MCI	55 subjects	24–30	0.5	A memory complaint Have objective memory loss measured by education adjusted scores on Wechsler Memory Scale 7 Logical Memory II Absence of significant levels of impairment in other cognitive domains, essentially preserved activities of daily living, and an absence of dementia
Normal	65 subjects	24–30	0	Non-depressed, non-MCI, and non-demented

Age ranged 56–80 years old.

**Table 3:** T_1_-weighted MRI scan parameters.

Dataset	ADNI
Slice thickness (mm)	1.2
No of slices	170
Scan matrix	256 × 256
TR (ms)	2300
TE	Minimum full TE
Pulse sequence	MPRGA
TI (ms)	900
FOV (cm)	26

According to the ADNI manual protocol the general inclusion/exclusion criteria points were developed to identify individuals with the amnestic form of MCI. Education-adjusted cut scores on logical memory were used to ensure that a true memory deficit existed and that the memory deficit was severe enough to ensure an adequate conversion rate to AD in the placebo-treated population. An adjudication committee was established to review data when a site believed that a subject had converted from MCI to AD. The general inclusion/exclusion criteria for all recruiters are as follows:

- All subjects must be willing and able to undergo all test procedures including neuroimaging and agree to longitudinal follow up. Specific psychoactive medications will be excluded. Exclusion for patients will be also applied if Any significant neurologic disease other than suspected incipient Alzheimer’s disease, such as Parkinson’s disease, multiple sclerosis, etc. or history of significant head trauma or known structural brain abnormalities. Any significant systemic illness or unstable medical condition which could lead to difficulty complying with the protocol.

All MRI and PET scans will be rapidly assessed for quality so that subjects may be rescanned if necessary. All clinical data will be collected, monitored, and stored by the Coordinating Center at UCSD. U Penn will collect biomarker samples. All raw and processed image data will be archived at LONI. Any more details about the inclusion/exclusion criteria can be found in the following link: https://adni.loni.usc.edu/wp-content/themes/freshnews-dev-v2/documents/clinical/ADNI-1_Protocol.pdf

### Data preparation and preprocessing

**Figure 1:**
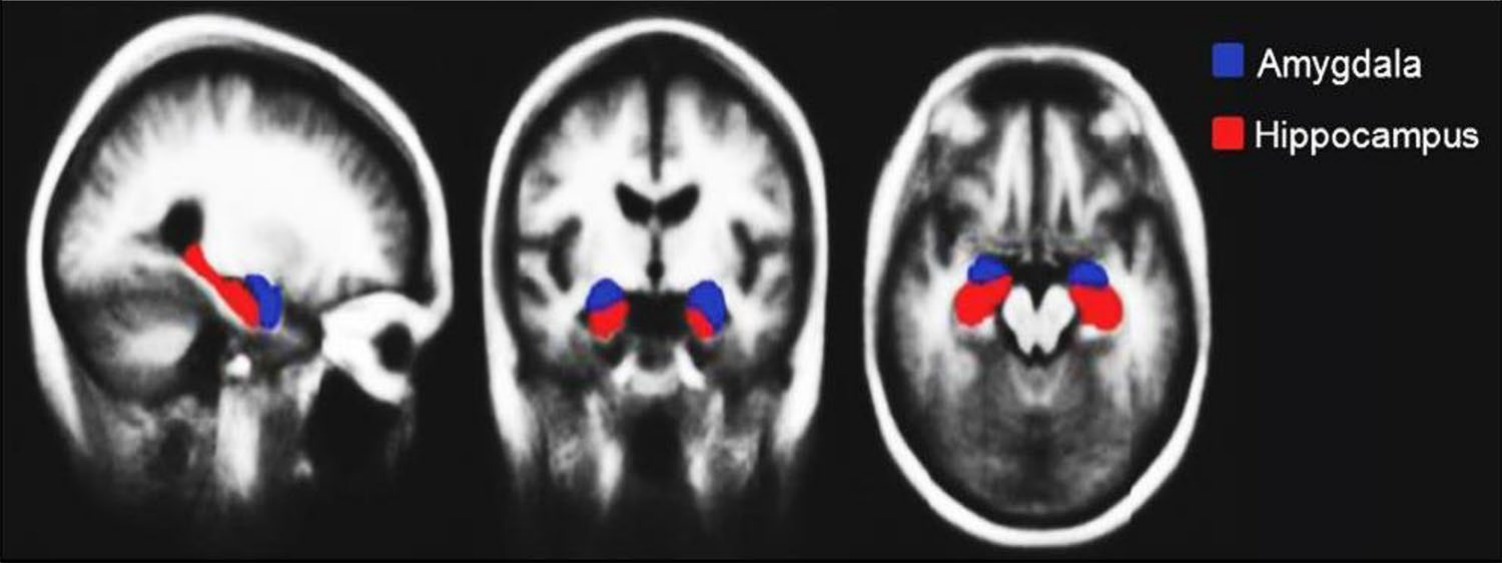
Hippocampus and amygdala position.

FMRIB Software Library (FSL)^26^, created at Oxford University, is a software library for image analysis and statistical tools for functional, structural, and diffusion MRI brain imaging data. Herein, to extract the Hippocampus and Amygdala, FSL applied through the following steps:

1. Brain Tissue Extraction: the goal of this step is to extract the brain from the skull using Brain Extraction Tool (BET) from FSL software. From an image of the whole head, BET deletes non-brain tissue. The inner, and outer skull surfaces can also be estimated, from good T_1_ and T_2_ images.
2. Registration to Atlas: this step is considered a crucial step for the correct segmentation of the ROIs, to overcome the differences in size and position of the brain.
3. Segmentation of the hippocampus, and amygdala.
4. Hippocampus and amygdala region fusion: this step is done using the FSL visualization tool, based on Harvard Oxford subcortical structure, as shown in Fig. 1. The region fusion is calculated using the Fslmath command to add up the extracted hippocampus to the extracted amygdala so that to form one ROI, then it is treated as a new region which will be named Hippo-Amygdala ROI later to be analyzed.

### Gauss map

**Figure 2:**
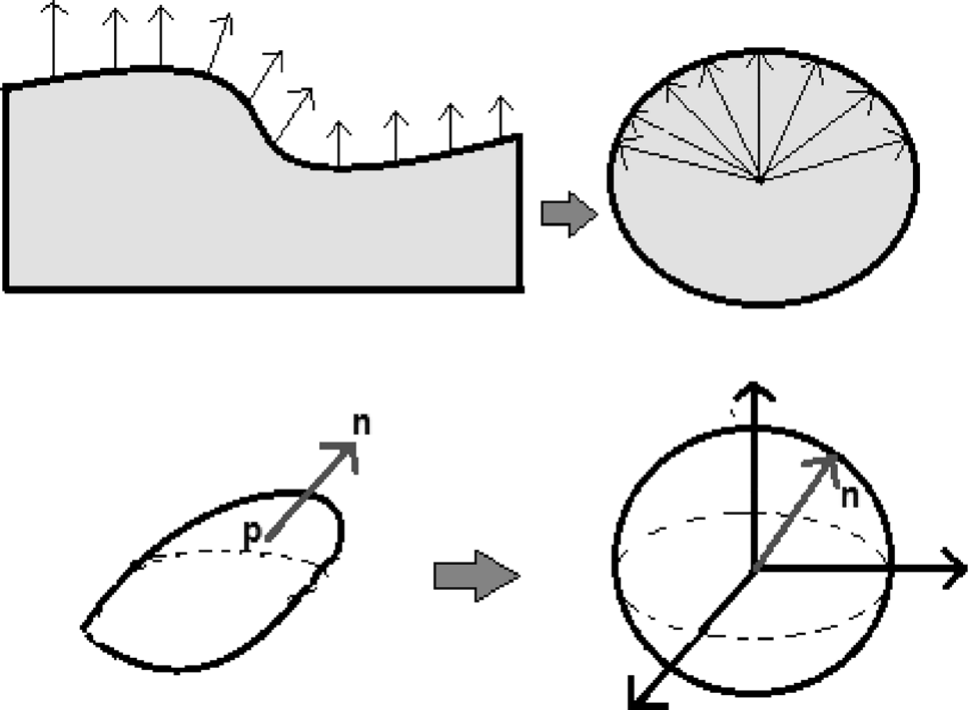
Gauss map definition.

In Euclidean space R^3^, the Gauss map depicts a surface to a unit sphere S^2^ while saving its shape. Consequently, the uniform sampling of points or vertices on the surface, the normal vector orientation of points is changed after rotation. Figure 2 shows the translation of the normal vector *n* of point *p*, from the original surface to the unit sphere, where its origin coexists with the origin of the coordinate. Gaussian mapping is the name of the process, and the sphere is known as the Gaussian sphere^27,28^.

**Figure 3:**
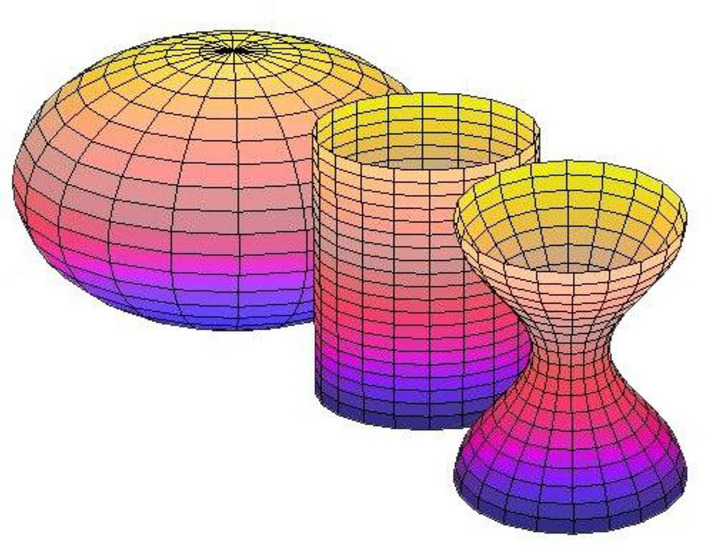
From right to left: negative Gaussian curvature is shown as hyperboloid surface, a surface of zero Gaussian curvature is a cylinder, and a positive Gaussian curvature surface is a sphere.

The variation of the normal vector *n,* at any point *p,* is measured using the derivative of the Gauss map at the same point *p*. Based on the Gauss Map, three main feature sets were employed, in this study, named the Gaussian shape operator, the Gaussian curvature, and the mean curvature. To measure how the surface bends, the Gaussian shape operator is constructed. It is dissipating the shape by erecting a matrix from normal and tangent at each point on the surface of the curve^29^. The eigenvalues of the shape operator is representing the maximum and minimum bending of the surface at the point *p*, well known by the principal curvatures ${\upkappa }_{1}$ and ${\upkappa }_{2}$_,_ measure. The dot product of this principle curvature, $K = {\upkappa }_{{1}} \cdot {\upkappa }_{{2}} ,$ is defined as the Gaussian curvature. The sign of the Gaussian curvature can be used to characterize the surface depending on the sign of the principal curvature. As shown in Fig. 3, If the principal curvatures have the same sign (${\upkappa }_{{1}} \cdot {\upkappa }_{{2}}$ > 0), then, the Gaussian curvature is positive and the corresponding surface is elliptic. If any of the principal curvatures is zero (${\upkappa }_{{1}} \cdot {\upkappa }_{{2}}$ = 0), then, the Gaussian curvature is zero and the expected surface should be following a parabolic. If the principal curvatures have different signs, the dot product is negative (${\upkappa }_{{1}} \cdot {\upkappa }_{{2}}$ < 0). Then, the Gaussian curvature is negative representing a hyperbolic or saddle point surface. Thus, the Gaussian curvature can help trace the hippocampus and amygdala curvature exchange from one point to another on the surface.

The mean curvature is the average of the signed curvature, as shown in the following equation:

1 $${\text{H}} = \frac{{{\upkappa }_{{1}} + {\upkappa }_{{2}} }}{2}$$

From the stated previous mathematical background, it shows that the Gaussian Map features were extracted for both the Hippocampus and Amygdala. This is to understand the overall changes in the shape of each than studying only the volume of each. The descriptors are spherical harmonic features widely used for describing and detecting objects in volumetric images in a rotational invariant manner it is not only included the signal from a single voxel but also includes the neighboring voxels which describes the pattern of the fibers distribution in the different brain regions for normals, MCIs, and ADs. This Hippocampus and Amygdala shape analysis may be more informative than global Hippocampus and Amygdala volume changes. The shape analysis can provide important information on the spatial distribution of atrophy or expansion of a structure that exhibits volume abnormalities and based on the substructures involved and their functional role may aid in understanding the functional outcomes of such volume abnormalities.

Makkinejad et al. examined the amygdala shape abnormalities associated with TDP-43 pathology in aging^31^. Transactive response DNA-binding protein 43 (TDP-43) pathology is common in old age and is strongly associated with cognitive decline and dementia. TDP-43 pathology has been reported in up to 55% of persons with Alzheimer’s disease (AD) pathology, TDP-43 pathology in aging has been shown to account for nearly as much of the variance of late-life cognitive decline as neurofibrillary tangles (hallmark pathology of AD)^31^. In persons with AD, the TDP-43 pathology is first deposited in the amygdala, followed by the hippocampus, and other regions^31^. Makkinejad et al. shape analysis revealed a unique pattern of amygdala deformation associated with TDP-43 pathology, adding up that the information from the ex vivo MRI can provide similar information on amygdala shape as that originating from in vivo MRI^31^.

As hippocampal pathology is viewed as a central landmark in AD, it has also been used as a diagnostic biomarker in clinical. The hippocampus composed of four sub regions which are CA1, CA2, CA3 and CA4^32^. Others study stated increased pathological burdens of P-Tau and P-Syn and associated microglia alterations are involved in a more severe deterioration of the CA1 in AD. A sub region that is effected during Alzheimer^33,34^.

While earlier volumetric MRI studies investigated pathology by measuring the total volume loss of hippocampus, recent studies attempt to identify regional pathology within the structure by analyzing the shape deformation of a particular sub-region, a method used in several studies of AD^35^. Each of the descriptors describing certain characteristics like the local curvature of the smoothed fiber distribution which influenced during the different stages of the disease as mentioned in the literature before^15,36^.The advantage of this approach that it models the data locally. Hence it can deal with the data where a significantly large areas differs from the prototype.

### Feature selection, classification, and performance evaluation

Dimensionality reduction is an important step, which helps to avoid an over fitting case since the quantum of features is considered huge concerning the size of data volumes used in this study. Moreover, the size of features is mainly based on the quantum of vertices representing the extracted volume, which is variable from one volume to another. Dimensionality reduction is appointed based on a well-known feature selection approach named Fisher Score^37,38^. The Fisher Information measures the amount of information that an observable random variable X carries about an unknown parameter θ of a distribution that models X^39^. As a classifier, Support Vector Machine (SVM) is employed as a supervised learning algorithm that analyzes data used for classification. Its goal is to separate between different classes by learning a function that is induced from available examples. SVMs can perform linear and non-linear classification. In this study, an SVM- Radial Basis Function (RBF) based kernel using LIBSVM was utilized, a package developed by the department of computer science of National Taiwan University^40^. To evaluate the system’s Performance, the Receiver operating characteristic (ROC) curve is employed since it functions well in assessing the diagnostic potency of tests to distinct the true state of subjects and comparing two stand by diagnostic tasks when each task is uttered on the same subject^27^.

### Approaches and experiments

Based on the medical understanding from the literature^9,15,34–36,41–45^, differences in the atrophy rates have been described in medial temporal lobe structures between patients with MCI and controls. Moreover, increased hippocampal deterioration rates have been found in patients with familial AD before clinical symptoms occur, and in addition more widespread declination in other cortical areas occur^9,36,40^. This pattern of prevalent atrophy is theoretically considered as an evident in patients with MCI later progressing to AD^40^. Moreover, this shrinkage was considered as a biomarker in previous studies such as Alzheimer detection, depression^9,41^. Thus this paper considered investigating the volume of hippocampus shrinkage during Alzheimer and its decrease with the deterioration of the disease by firstly experimenting the hippocampal atrophy before using the Gaussian map descriptors to differentiate between normal, AD and MCI, results of this experiment will presented in the following section. Since hippocampus volumetry calculation and segmentation is a great problem and many tools were introduced to calculate it such as freesurfer, open source VolBrain. This article used the later tool (an open-source tool)^42^, was developed for hippocampus T_1_-weighted MRI images, to segment and calculate the volume of different regions in the brain.

Thus the other method employed based on the Gaussian Map based features, extracted from ROI to distinguish between the normal controls, MCI, and AD patients. The Gaussian Map features were extracted for both the Hippocampus and Amygdala separately once and once again after contaminating the two regions to study the overall changes in the shape than studying this for each region separately. For more assessment and understanding, two fusion levels were done; Feature-fusion level which was done by calculating independently each ROI extracted feature and then fusing both for further classification. ROI-fusion level whereas fusion of the regions before feature extraction was also employed. The classification process is following hierarchical criteria where, in the first phase, the objective is to discriminate NC from the abnormal subjects; named AD and MCI. The second step is to differentiate the AD and MCI subjects. The Gaussian-based extracted features were then given to the SVM classifier. Finally, the performance of the proposed extracted features will be evaluated calculating sensitivity, specificity, accuracy, and ROC analysis.

## Results and discussion

Table 4 shows the results of our first experiment that investigates the hippocampal, amygdala atrophy to differentiate between normal, AD and MCI. Open source VolBrain tool was used to segment and calculate the volume of different regions in the brain. The classification in between normal, MCI and AD based on the hippocampus, and the amygdala atrophy.

**Table 4:** Accuracies of classifier based on volumes of ROI.

Feature type	Normal vs abnormal (%)	AD vs MCI (%)
Amygdala atrophy	68	60
Hippocampus atrophy	72	60.5
Feature level fusion	65.2	67.8
Region level fusion	62.6	65.9

From Table 4 we can conclude that although the results were acceptable but still there are better results that can be achieved by calculating the Gaussian map descriptor features as it is depending not only on the volume size but also on the shape of the brain region. This result agrees with what Ahmed et al.^15^ presented in her research. They did automatically categorize individuals with AD and/or MCI from anatomical MRI using several methods. Depending on which MRI features are extracted, these methods can be loosely divided into three categories: voxel-based, vertex-based, or ROI-based analysis^39^. Additionally, based on the results of her survey, ROI-based analysis suggests that shape rather than volume is more significant in the hippocampus.

Although, Henneman et al.^44^ stated there are differences in the rates of atrophy (atrophy) in the medial temporal lobe structures between MCI patients and controls. Even before clinical symptoms appear, persons with familial AD had higher hippocampus shrinkage rates. More extensive atrophy in other cortical regions occurs in AD patients. Patients with MCI that subsequently advance to AD already show this pattern of extensive atrophy. We demonstrate that, contrary to what has previously been reported, hippocampus shrinkage (rate) does not distinguish patients with AD from patients with MCI. This confirms past research showing that the rate of hippocampus shrinkage resembling AD is already present during the transitional stage (MCI). After this point, whole brain atrophy rates, which continue to rise with increasing disease severity, become a more accurate indicator of disease progression than assessments of hippocampal volume.

**Figure 4:**
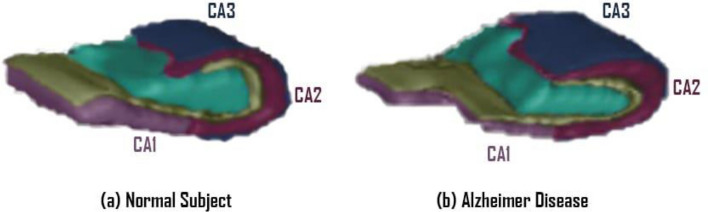
The hippocampus composed of four sub regions which are CA1, CA2, CA3 and CA4, and the hippocampus erosion occurred due to the Alzheimer disease (**b**) compared with the normal subjects (**a**).

In more details, the hippocampus composed of four sub regions which are CA1, CA2, CA3 and CA4^9^ as shown in Fig. 4. The sub region that have been effected during Alzheimer is CA1. CA1 curve shape has been shrinkage so changes in its curvature with respect to shape will be a great indicator of Alzheimer detection. Mean curvature is the average of the signed curvature. As illustrated, calculating the average will not be sufficient enough as change happened through the same direction and a small area compared with the hippocampus overall^41^.

According to Mueller et al.^45^, normal ageing and AD, even in its initial phases, are linked to a distinctive pattern of hippocampal atrophy, i.e. ageing with volume loss in CA1 and MCI with volume loss in CA1-2 transition. Volume loss in the CA1-2 transition outperformed total hippocampus volume in terms of separating subjects with MCI from controls. In light of this, they draw the conclusion that subfield measures may be a more accurate method of identifying MCI and early-stage AD than measurements of the entire hippocampus.

From the previous discussion that shows that hippocampal atrophy (rate) was not enough to differentiate patients with AD from patients with MCI, as has also been reported by others. To discriminate between AD, MCI, and Normal cases, we applied primarily Gaussian map descriptor features such as shape operator, Gaussian curvature, and mean curvature on three ROIs (Hippocampus, Amygdala, and Hippo-Amygdala region based fusion”) separately, Region-based feature-based fusion was also calculated. Next, these features were fed to the SVM classifier. The Classification task was implemented through a hierarchical framework. The first step is discriminating between Normal and Abnormal cases, which includes both AD and MCI subjects. The second step is classifying the abnormal cases into AD and MCI. Table 5 shows the classifier accuracy owing to Gaussian map features for region fusion which shows that the shape operator gives the result of 74.6% for distinguishing between normal and abnormal cases with a significance p-value equal to 0.05, while Gaussian curvature and mean curvature give 72.2% and 73.9% respectively. All Gaussian map descriptors (shape operator, Gaussian curvature, and mean curvature) features give about 98.9%, 98.5%, and 98.2% of distinguishing between AD and MCI with significance p-value equal to 0.1, 0.3, and 0.1 respectively. The good significant results make the proposed system to be more stable and robust. While using the features of the hippocampus region only for normal/abnormal classification, it gives about 73.2%, 69.5%, and 61% respectively for shape operator, Gaussian curvature, and mean curvature, Table 6.

**Table 5:** The classifier accuracy owed to Gauss map features for hippocampus and amygdala region level fusion, and for hippocampus features and amygdala features level fusion respectively.

Feature type	Region level fusion			Feature level fusion		
	Normal vs abnormal (%)	AD vs MCI (%)	Overall accuracy (%)	Normal Vs abnormal (%)	AD vs MCI (%)	Overall accuracy (%)
Shape operator	74.6	98.9	82.67	73.4	53.8	64.8
Gaussian curvature	72.2	98.5	82.6	70.4	96.2	80.6
Mean curvature	73.9	98.2	82.35	60.9	58.5	58.4

**Table 6:** The accuracy of the classifier for Gauss map features for the hippocampus and amygdala regions separately.

Feature type	Hippocampus			Amygdala		
	Normal vs abnormal (%)	AD vs MCI (%)	Overall accuracy (%)	Normal vs abnormal (%)	AD vs MCI (%)	Overall accuracy (%)
Shape operator	73.2	58.99	65.6	60.7	53.8	58.19
Gaussian curvature	69.5	98.3	81.11	65.4	54	58.13
Mean curvature	60.8	50.7	58.3	68	49.6	58.11

Fusion of the regions (hippocampus, amygdala), enlarging the ROI make the system able to measure the curvatures changes better, consequently, the results were improved. Table 5 shows the accuracy of the classifier for Gauss Map features for features fusion which shows that the shape operator gives a result of 73.4% for distinguishing between normal and abnormal cases with a p-value equal to 0.14 to of significance while the Gaussian curvature feature gives about 96.2% of distinguishing between AD and MCI with a p-value equal to 0.2 of significance.

The entire volume of the hippocampus shrinks as the disease progresses, it serves as a strong indication of AD. Subcortical structure segmentation using VolBrain produces different results for normal, MCI, and AD brains. The accuracy of Gauss Map features (Shape) for the Amygdala area is shown to be lower than that determined based on the volume calculation when calculating the classifier's accuracy. A different example is the hippocampal region, where Gauss Map-based features perform better than those based on volume calculations, which makes sense given that shape provides more information than volume. Additionally, regardless of the disease stage, there is intra-class variability, meaning that each patient's brain volume varies.

Since, in the belated terms of AD, an utmost attrition in the shape and the curvature of the Hippocampus. It can be used to describe the progression of the disease^6,9,16,20,34^. Hippocampus and amygdala curve shape have been shrinkage so changes in its curvature from one point to another on its contour surface concerning shape will be a great indicator of Alzheimer’s detection. Though, a key solution might by characterizing this change by extracting new features.

To study the robustness of the system, ROC curves for each classifier under investigation as well as the area under the curves are calculated. The Amygdala region is very small and after its segmentation, it was blurred and has little information so its performance was very bad as its accuracies vary between 48 and 52% for the three features.

**Figure 5:**
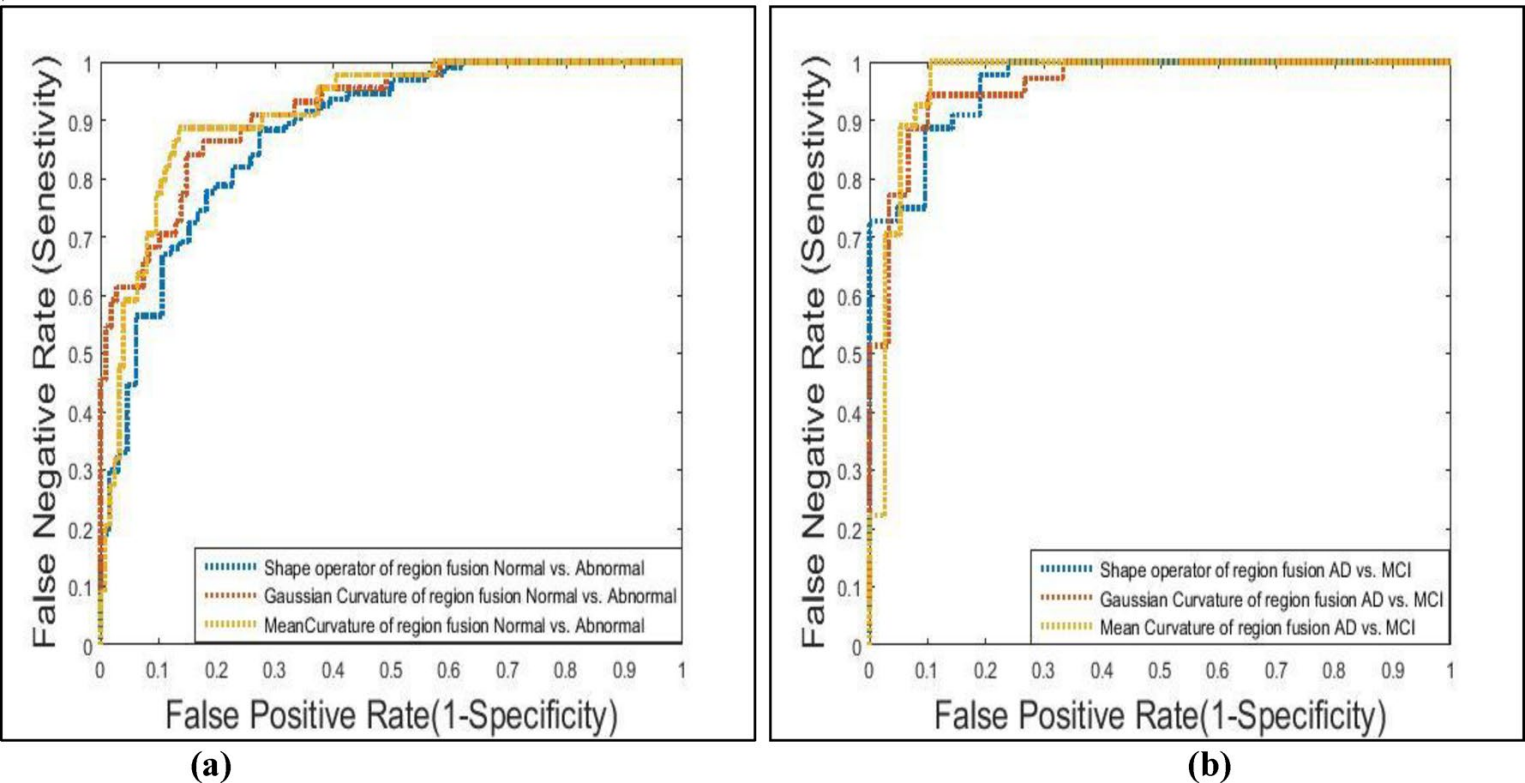
Gaussian map descriptors features ROC curve for Hippo-Amygdala region fusion for: (**a**) normal vs. abnormal, (**b**) AD vs. MCI.

**Figure 6:**
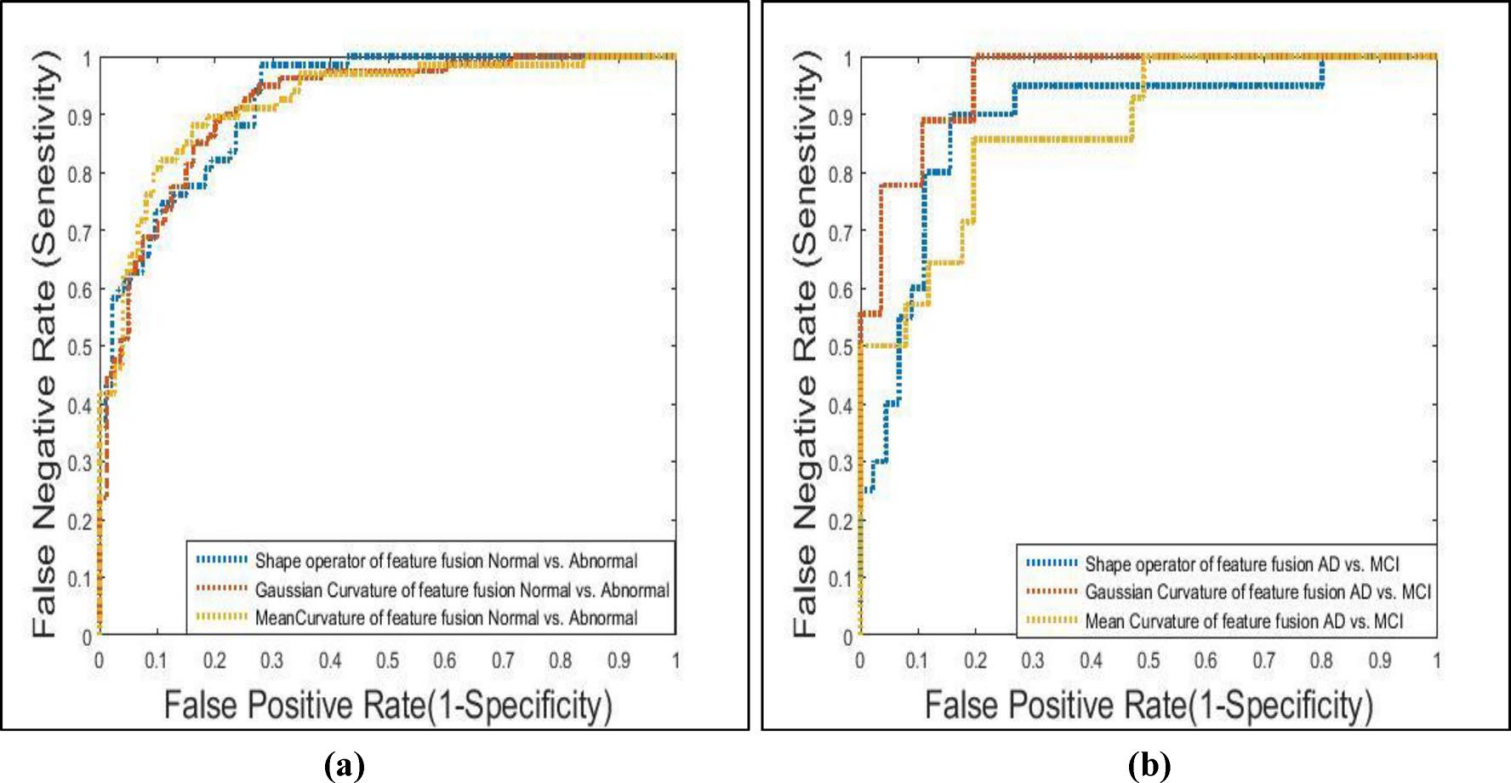
Gaussian map descriptors features ROC curve for hippocampus amygdala feature fusion for: (**a**) normal vs. abnormal, (**b**) AD vs. MCI.

Based on Fig. 5a, it is observed that in the case of normal and abnormal classification for Hippo-Amygdala ROI, mean curvature and Gaussian curvature have a roughly equal ability of performance, with an AUC of 0.773 and 0.754 respectively. In the case of the AD vs. MCI classifier, Based on Fig. 5b, the shape operator, mean curvature and Gaussian curvature have roughly equal stability of performance, as AUC equal roughly 0.98, 0.975, and 0.97 respectively. Figure 6a shows that in the case of normal and abnormal classification using feature fusion, the mean curvature, Gaussian curvature, and shape operator have roughly equal stability of performance, with an AUC of 0.786, 0.783, and 0.781 respectively. Whereas, in the case of AD vs. MCI classifier, the Gaussian curvature performed better than the other Gaussian map descriptors features, as AUC was equal to roughly 0.98, 0.83, and 0.76 for the Gaussian curvature, Gaussian Shape operator and mean curvature respectively, as shown in Fig. 6b.

## Conclusion

This manuscript, features extracted representing Gaussian-based descriptors were appointed to differentiate the normal, from AD, and MCI subjects based on dissecting the shape of the hippocampus and Amygdala. FSL was used for brain extraction, registration, and segmentation of the hippocampus and amygdala then fusion between these two regions was done. Three different feature sets were extracted name: the Gaussian Shape operator, the Gaussian curvature, and the mean curvatures. These features are calculated for each of the hippocampus and amygdala individually. Moreover, the Features extracted for each region were fused once and the features extracted from Hippo-Amygdala based on region fusion were employed once more. When classifying the AD from MCI, results are deemed very promising reaching 98% accuracy using region fusion Gaussian shape operators, while the performance is as yet affronting in the case of normal/abnormal classification to attain 74.6% and 73.3% using the Gaussian Shape operator based system for region fusion and feature fusion respectively. Given that the increased hippocampal deterioration rates can be used to classify the various stages of illness progression and analyze the structure of the regions, the Gauss map is thought to be a very promising tool. The implemented research work still needs to include the following research points:

1. Growing the dataset would unquestionably provide us more space to investigate the system's robustness (limitation)
2. More research needs to be done on the proposed qualities combined in a hierarchical or perhaps a three-class system.
3. We can use both the shape and the texture-based features in the study because we did not analyze the texture-based characteristics at all.

In conclusion, the proposed system is considered promising. Nevertheless, the combination of the proposed features in hierarchical or even in a three-class system needs to be further investigates. Also the volume of these ROI may be added as a fourth features that can be used.

## Data Availability

The dataset used in this study was obtained from a third-party organization “Alzheimer's disease Neuroimaging Initiative” (ADNI) database. The data are available from the ADNI database (adni.loni.usc.edu) upon registration and compliance with the data usage agreement. For up-to-date information, see www.adni-info.org. The proposed algorithm uses this ADNI data repository. The data that support the findings of this study are available on reasonable request from the authors. All ADNI studies are conducted according to the Good Clinical Practice guidelines, the Declaration of Helsinki, and U.S. 21 CFR Part 50 (Protection of Human Subjects), and Part 56 (Institutional Review Boards). Written informed consent was obtained from all participants before protocol-specific procedures were performed. The ADNI protocol was approved by the Institutional Review Boards of all of the participating institutions. This study was approved by the Institutional Review Boards of all of the participating institutions, such as the Office for the Protection of Research Subjects at the University of Southern California. A complete listing of ADNI investigators and affiliations can be found at http://adni.loni.usc.edu/wp-content/uploads/how_to_apply/ADNI_Acknowledgement_List.pdf. Informed written consent was obtained from all participants at each site. The investigators within the ADNI contributed to the design and implementation of ADNI and/or provided data but did not participate in analysis or writing of this report. More details can be found at adni.loni.usc.edu.

## References

[1] Murphy SL, Xu J, Kochanek KD, Curtin SC, Arias E (2017). Deaths: Final data for 2015. Natl. Vital Stat. Rep..

[2] Asscociation TA (2022). 2022 Alzheimer’s disease facts and figures. Alzheimer’s Dement..

[3] Alzheimer’s Disease: Causes, Symptoms and Treatments. (https://www.mayoclinic.org/diseases-conditions/alzheimers-disease/symptoms-causes/syc-20350447).

[4] Johnson KA, Fox NC, Sperling RA, Klunk WE (2012). Brain imaging in Alzheimer disease. Cold Spring Harb. Perspect. Med..

[5] Liu Z, Lu H, Pan X, Xu M, Lan R, Luo X (2022). Diagnosis of Alzheimer’s disease via an attention-based multi-scale convolutional neural network. Knowl. Based Syst..

[6] Hazarika RA, Maji AK, Sur SN, Paul BS, Kandar D (2021). A Survey on classification algorithms of brain images in Alzheimer’s disease based on feature extraction techniques. IEEE Access.

[7] Amoroso N, La Rocca M, Bellotti R, Fanizzi A, Monaco A, Tangaro S (2018). Alzheimer’s disease diagnosis based on the Hippocampal Unified Multi-Atlas Network (HUMAN) algorithm. Biomed. Eng. Online.

[8] Nie X (2017). Subregional structural alterations in hippocampus and nucleus accumbens correlate with the clinical impairment in patients with Alzheimer’s Disease clinical spectrum: Parallel combining volume and vertex-based approach. Front. Neurol..

[9] Boutet C (2014). Detection of volume loss in hippocampal layers in Alzheimer’s disease using 7 T MRI: A feasibility study. NeuroImage Clin..

[10] Damulina A (2019). White matter hyperintensities in Alzheimer’s disease: A lesion probability mapping study. J. Alzheimer’s Dis..

[11] Fan Y, Shen D, Gur RC, Gur RE, Davatzikos C (2007). COMPARE: classification of morphological patterns using adaptive regional elements. Comp. A J. Comp. Educ..

[12] Gerardin E (2009). Multidimensional classification of hippocampal shape features discriminates Alzheimer’s disease and mild cognitive impairment from normal aging. Neuroimage.

[13] Chupin M (2009). Fully automatic hippocampus segmentation and classification in Alzheimer’s disease and mild cognitive impairment applied on data from ADNI. Hippocampus.

[14] Chupin M (2009). Automatic segmentation of the hippocampus and the amygdala driven by hybrid constraints: Method and validation. Neuroimage.

[15] Ben Ahmed O, Mizotin M, Benois-Pineau J, Allard M, Catheline G, Ben Amar C (2015). Alzheimer’s disease diagnosis on structural MR images using circular harmonic functions descriptors on hippocampus and posterior cingulate cortex. Comput. Med. Imaging Graph.

[16] Rathore S, Habes M, Iftikhar MA, Shacklett A, Davatzikos C (2017). A review on neuroimaging-based classification studies and associated feature extraction methods for Alzheimer’s disease and its prodromal stages. Neuroimage.

[17] Lotterie J (2009). Early diagnosis of Alzheimer’s disease using cortical thickness: Impact of cognitive reserve. Brain.

[18] Rathore S, Habes M (2017). A review on neuroimaging-based classification studies and associated feature extraction methods for Alzheimer’s disease and its prodromal stages. Neuroimage.

[19] Magnin B (2009). Support vector machine-based classification of Alzheimer’s disease from whole-brain anatomical MRI. Neuroradiology.

[20] Mousa D, Zayed N, Yassine IA (2022). Alzheimer disease stages identification based on correlation transfer function system using resting-state functional magnetic resonance imaging. PLoS ONE.

[21] Hartley, T., Bird, C. M., Chan, D., Cipolotti, L., Husain, M., & Burgess, N. Europe PMC Funders Group The hippocampus is required for short-term topographical memory in humans. **17**(1), 34–48. 10.1002/hipo.20240 (2009). 10.1002/hipo.20240 PMC2677717 17143905

[22] Bird CM, Burgess N (2008). The hippocampus and memory: Insights from spatial processing. Nat. Rev. Neurosci..

[23] Aranzi JC, German T (2022). Hippocampus cognitive map. Hippocampus.

[24] Baxter MG, Croxson PL (2012). Facing the role of the amygdala in emotional information processing. Proc. Natl. Acad. Sci. USA.

[25] Hajian-Tilaki K (2013). Receiver operating characteristic (ROC) curve analysis for medical diagnostic test evaluation. Casp. J. Intern. Med..

[26] Jenkinson M, Beckmann CF, Behrens TEJ, Woolrich MW, Smith SM (2012). Fsl. Neuroimage.

[27] Pan J, Luo H, Lu Z, Chang J (2006). A new 3D shape descriptor based on rotation. Sixth Int. Conf. Intell. Syst. Des. Appl..

[28] Gray, S., Abbena, E., & Salamon. *Modern Differential Geometry of Curves and Surfaces with Mathematica*, (3rd ed.). Chapman and Hall/CRC., 2006. [Online]. Available: 10.1201/9781315276038.

[29] Elsa Abbena, A. G., & Salamon, S. Shape and curvature. In *Modern Differential Geometry of curves and surfaces with Mathematica*, Third Edit., pp. 385–419.

[30] Skibbe, H., Reisert, M., & Burkhardt, H. Gaussian neighborhood descriptors for brain segmentation. MVA (2011).

[31] Makkinejad N, Schneider JA, Yu J, Leurgans SE, Kotrotsou A, Evia AM, Bennett DA, Arfanakis K (2019). Associations of amygdala volume and shape with trans-active response DNA-binding protein 43 (TDP-43) pathology in a community cohort of older adults. Neurobiol. Aging..

[32] Anand KS, Dhikav V (2012). Hippocampus in health and disease: An overview. Annu. Indian Acad. Neurol..

[33] Fixemer, S., *et al.* Concomitant AD and DLB pathologies shape subfield microglia responses in the hippocampus bioRxiv, p. 2022.01.06.475218 (2022).

[34] Lindberg O, Walterfang M, Looi JC, Malykhin N, Ostberg P, Zandbelt B, Styner M, Paniagua B, Velakoulis D, Orndahl E, Wahlund LO (2012). Hippocampal shape analysis in Alzheimer's disease and frontotemporal lobar degeneration subtypes. J. Alzheimers Dis..

[35] Apostolova LG, Thompson PM, Green AE, Hwang KS, Zoumalan C, Jack CR, Harvey DJ, Petersen RC, Thal LJ, Aisen PS, Toga AW, Cummings JL, Decarli CS (2010). 3D comparison of low, intermediate, and advanced hippocampal atrophy in MCI. Hum. Brain Mapp..

[36] Abdelaziz M, Wang T, Elazab A (2021). Alzheimer’s disease diagnosis framework from incomplete multimodal data using convolutional neural networks. J. Biomed. Inform..

[37] Li C, Xu J (2019). Feature selection with the Fisher score followed by the Maximal Clique Centrality algorithm can accurately identify the hub genes of hepatocellular carcinoma. Sci. Rep..

[38] Zhen X, Shao L (2016). Action recognition via spatio-temporal local features: A comprehensive study. Image Vis. Comput..

[39] Gu, Q. Generalized Fisher score for feature selection a brief review of fisher score.

[40] Chang C, Lin C (2013). LIBSVM: A library for support vector machines. ACM Trans. Intell. Syst. Technol..

[41] Sapolsky RM (2001). Depression, antidepressants, and the shrinking hippocampus. Proc. Natl. Acad. Sci..

[42] Manjón JV, Coupé P (2016). volBrain: An online MRI brain volumetry system. Front. Neuroinf..

[43] Cuingnet R (2011). Automatic classification of patients with Alzheimer’s disease from structural MRI: A comparison of ten methods using the ADNI database. Neuroimage.

[44] Henneman WJP (2009). Hippocampal atrophy rates in Alzheimer disease: Added value over whole brain volume measures. Neurology.

[45] Mueller SG, Schuff N, Yaffe K, Madison C, Miller B, Weiner MW (2010). Hippocampal atrophy patterns in mild cognitive impairment and Alzheimer’s disease. Hum. Brain Mapp..

